# In-Hospital Cardiac Arrest in United States Emergency Departments, 2010–2018

**DOI:** 10.3389/fcvm.2022.874461

**Published:** 2022-04-11

**Authors:** Chih-Wei Sung, Tsung-Chien Lu, Chih-Hung Wang, Eric H. Chou, Chia-Hsin Ko, Chien-Hua Huang, Wen-Jone Chen, Chu-Lin Tsai

**Affiliations:** ^1^Department of Emergency Medicine, National Taiwan University Hospital Hsin-Chu Branch, Hsinchu, Taiwan; ^2^Department of Emergency Medicine, National Taiwan University Hospital, Taipei, Taiwan; ^3^Department of Emergency Medicine, College of Medicine, National Taiwan University, Taipei, Taiwan; ^4^Department of Emergency Medicine, Baylor Scott and White All Saints Medical Center, Fort Worth, TX, United States

**Keywords:** emergency department, incidence, mortality, trend, in-hospital cardiac arrest

## Abstract

**Background:**

Little is known about the in-hospital cardiac arrest (IHCA) in the US emergency department (ED). This study aimed to describe the incidence and mortality of ED-based IHCA visits and to investigate the factors associated with higher incidence and poor outcomes of IHCA.

**Materials and Methods:**

Data were obtained from the National Hospital Ambulatory Medical Care Survey (NHAMCS) between 2010 and 2018. Adult ED visits with IHCA were identified using the cardiopulmonary resuscitation code, excluding those with out-of-hospital cardiac arrest. We used descriptive statistics and multivariable logistic regression accounting for NHAMCS’s complex survey design. The primary outcome measures were ED-based IHCA incidence rates and ED-based IHCA mortality.

**Results:**

Over the 9-year study period, there were approximately 1,114,000 ED visits with IHCA. The proportion of IHCA visits in the entire ED population (incidence rate, 1.2 per 1,000 ED visits) appeared stable. The mean age of patients who visited the ED with IHCA was 60 years, and 65% were men. Older age, male, arrival by ambulance, and being uninsured independently predicted a higher incidence of ED-based IHCA. Approximately 51% of IHCA died in the ED, and the trend remained stable. Arrival by ambulance, nighttime, or weekend arrival, and being in the non-Northeast were independently associated with a higher mortality rate after IHCA.

**Conclusion:**

The high burden of ED visits with IHCA persisted through 2010–2018. Additionally, ED-based IHCA survival to hospital admission remained poor. Some patients were disproportionately affected, and certain contextual factors were associated with a poorer outcome.

## Introduction

In-hospital cardiac arrest (IHCA) is associated with high morbidity and mortality worldwide. In the United States, there have been approximately 200,000 IHCA patients annually between 2003 and 2007 (1). This number has increased to 300,000 each year between 2013 and 2018 (2). The estimated incidence of IHCA varies by geography and demographics, ranging from 1.3–6 per 1,000 hospital admissions in Australia and New Zealand (3) to 9–10 per 1,000 in the United States (2).

With advances in emergency and critical care medicine, IHCA survival-to-discharge rates appear to be improving but plateaued recently (4–6). However, there is still room for improvement regarding post-IHCA survival. Recent studies have reported survival-to-hospital discharge is only 20–25% (7, 8). Moreover, a comprehensive understanding of potential risk factors for mortality in IHCA patients, including regional and hospital-level contextual factors, deserves more investigations (8).

Emergency department (ED)-based IHCA is a subset of IHCA that accounts for approximately 10% of cases (9). As previous studies focused on the entire IHCA population, the survival trends in ED-based IHCA and factors associated with survival-to-hospital admission (vs. survival-to-discharge) remain unknown. Valderrama et al. have analyzed the National Hospital Ambulatory Medical Care Survey (NHAMCS) data to investigate ED cardiac arrest (EDCA) in the US from 2001 to 2007. They found that the incidence rate of EDCA is approximately 1 in 600 ED visits. Moreover, men, the elderly, and uninsured individuals are more vulnerable to EDCA (10). However, that study examined EDCA that combined out-of-hospital CA (OHCA) and IHCA; whether these findings hold in ED-based IHCA remains unknown. Furthermore, this study did not examine trends in ED-based IHCA over time, nor did it investigate factors associated with survival to hospital admission in ED-based IHCA.

The current study aimed to describe the incidence and mortality of ED-based IHCA visits from the NHAMCS between 2010 and 2018. Further, we sought to investigate the potential factors associated with the incidence of ED-based IHCA and post-IHCA ED mortality.

## Materials and Methods

### Study Design and Setting

The NHAMCS is a cross-sectional, multistage probability sample of visits to non-institutional general and short-stay hospitals, excluding federal, military, and Veterans Administration hospitals, located in the 50 states and the District of Columbia (11). The NHAMCS is conducted annually by the National Center for Health Statistics (NCHS). It covers geographic primary sampling units, hospitals within primary sampling units, EDs within hospitals, and patients within EDs. The number of EDs sampled is approximately 300–400 per year. Trained ED staff collected clinical information during a randomly assigned 4-week period for each of the sampled EDs using a structured Patient Record Form (PRF). Data included patient demographics, reasons for the visit, diagnoses, procedures, medications given at the visit, and the basic characteristics of the treating physician and hospital. Quality control was performed using a two-way independent verification procedure of a 10% sample of the records. The non-response rate for most items was < 5%. The coding error rates were < 2% (12). Because the NHAMCS contains publicly available, de-identified data, the National Taiwan University Hospital Institutional Review Board exempted this study from review.

### Study Population

NHAMCS data from 2010 to 2018 were used in this analysis. First, we excluded ED visits from patients aged < 18 years. Next, we excluded patient visits due to OHCA (marked as “dead on arrival” on the PRF) as this analysis focused on IHCA. Adult ED visits with treated IHCA were identified using the cardiopulmonary resuscitation (CPR) procedure mark on the PRF.

### Variables

To preserve consistency across years, race/ethnicity was recoded as non-Hispanic white, non-Hispanic black, Hispanic, and other. Insurance was recoded as private, Medicare, Medicaid or other state-based programs, self-pay, and other. The US regions represented standardized geographical divisions, as defined by the US Census Bureau (Northeast, Midwest, South, and West) (13). Up to five reasons for each ED visit were coded using the Reason for Visit Classification for Ambulatory Care, a standardized sourcebook used in NCHS studies (14). The three most common reasons for the ED visit were reported. Chronic comorbid conditions were ascertained based on the PRF, including but not limited to diabetes mellitus, hypertension, coronary artery disease, chronic obstructive pulmonary disease, and cancer. Data on disease severity/urgency included triage levels, vital signs at triage, and pain scores. Visit disposition was recorded for each ED visit. For ED visits resulting in hospitalizations, inpatient mortality, and hospital length of stay (LOS) were recorded.

### Outcome Measures

The primary outcome measure was the ED-based IHCA incidence (visit) rate. We calculated this as the number of ED-based IHCA visits divided by the total adult ED population number, as described previously in the consensus guidelines on reporting IHCA (15). ED-based IHCA rates were reported per 1,000 visits per year. The co-primary outcome measure was ED-based IHCA mortality.

### Statistical Analysis

Stata 16.0 (StataCorp, College Station, TX) was used to adjust the variances to account for the complex design of the NHAMCS survey. Standard errors (SEs) were calculated for the NHAMCS estimates using the Stata svy commands. Statistical tests were generally based on estimates that had at least 30 cases and a less than 30% relative SE (i.e., the SE divided by the estimate expressed as a percentage of the estimate) in the sample data, according to the NCHS recommendations. For the mortality trend analysis, we combined 2 years of data to increase the stability of the estimates. Descriptive statistics were presented as proportions [with 95% confidence intervals (CIs)] or means (with SEs). We used the weighted chi-square test to analyze differences between proportions. Logistic regression models were used to test for significant changes in the primary outcomes (ED-based IHCA rates and ED mortality) during the study period, in which calendar year was a linear independent variable. Multivariable logistic regression analysis was performed to assess the independent predictors of the two primary outcomes. Due to the limited number of outcomes, the parsimonious multivariable models included age, sex, race/ethnicity, insurance, season, weekend, time of presentation, region, and arrival mode. We conducted two alternative analyses. First, we employed Poisson regression modeling to derive incidence rate ratios (IRRs) for IHCA occurrence. Second, we built clinical models including limited clinical information at ED triage for IHCA occurrence and mortality. Odds ratios (ORs) are presented with 95% CI. All *P*-values are two-sided, with *P* < 0.05 considered statistically significant.

## Results

**FIGURE 1:**
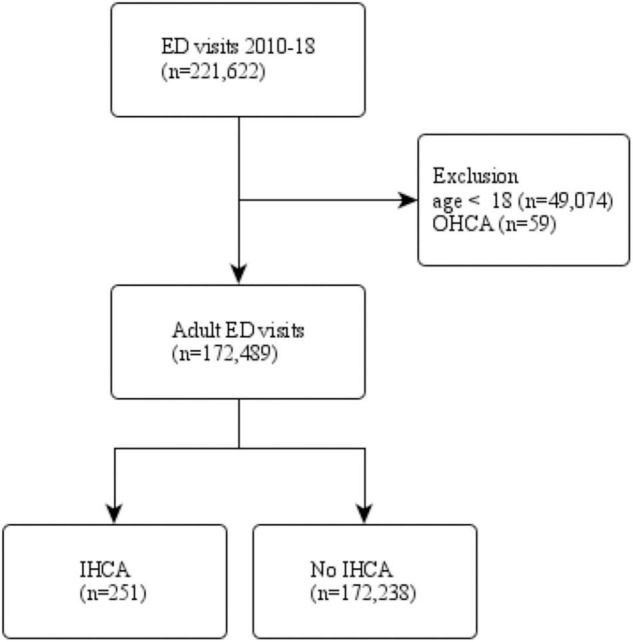
The patient selection process.

Figure 1 shows the patient selection process. There were 221,622 patients who visited ED between 2010 and 2018. After weighting, the study population represented 946,000,000 ED visits over the 9-year period. Non-adult patients and those with OHCA were excluded. A total of 172,489 adult ED patients were divided into two groups by IHCA status. Of them, 251 patients had IHCA during the ED stay.

Table 1 describes the baseline clinical characteristics of ED patients with IHCA. After weighting, 1,114,000 ED-based IHCA occurred during the study period. Nearly half of the patients were elderly, with predominantly male patients. Approximately half of the patients were non-Hispanic white, 24% non-Hispanic black, and 18% Hispanic. Nearly half of the patients had Medicare insurance, and others reported having private insurance (16%), self-pay (17%), or Medicaid (13%). ED-based IHCA occurrence was slightly higher in the fall than in other seasons. About 22% of ED-based IHCA occurred on the weekend, and most ED-based IHCA occurred in the daytime or evening. Hospitals in the South had nearly half of the ED-based IHCAs, while hospitals in other regions had fewer IHCA events. Most of the patients with IHCA later in the ED were sent by ambulance. The mean number of comorbid conditions was 1.8. The most common presenting symptoms included shock, chest pain, and shortness of breath. The ED-based IHCA events were triaged mostly into level 1 (54%), followed by levels 2 and 3. The mean length of ED stay was 7 h, with nearly half of the patients surviving to hospital admission. Of these, half died during hospitalization. The mean length of hospital stay was approximately 6 days.

**TABLE 1:** Baseline clinical characteristics of emergency department patients with in-hospital cardiac arrest, 2010–2018.

Variable	Weighted number, or weighted mean	Weighted percentage (95% CI)
Overall	1,114,000	
**Section A. Demographics and contextual information**		
**Age group**		
18–24	42,000	3.8 (2.1–6.8)
25–44	192,000	17.2 (11.7–24.7)
45–64	369,000	33.1 (25.6–41.6)
65–74	231,000	20.7 (15.2–27.6)
75 +	280,000	25.1 (18.5–33.1)
**Sex**		
Male	721,000	64.7 (56.6–72.1)
Female	393,000	35.3 (27.9–43.3)
**Race/ethnicity**		
Non-Hispanic White	600,000	53.8 (39.5–67.5)
Non-Hispanic Black	267,000	24.0 (17.3–32.2)
Hispanic	203,000	18.2 (7.1–39.4)
Other	44,000	4.0 (1.5–10.4)
**Insurance**		
Private insurance	166,000	16.6 (10.8–24.8)
Medicare	487,000	48.6 (38.0–59.4)
Medicaid or state-based program	129,000	12.9 (8.4–19.2)
Self-pay (uninsured)	172,000	17.2 (11.7–24.4)
Other	47,000	4.7 (2.1–9.9)
**Season**		
Spring (Mar. – May)	286,000	25.6 (15.3–39.7)
Summer (Jun. – Aug.)	228,000	20.4 (14.0–28.9)
Fall (Sep. – Nov.)	369,000	33.1 (23.3–44.6)
Winter (Dec. – Feb.)	232,000	20.9 (14.8–28.5)
Weekend	240,000	21.6 (15.8–28.8)
**Time of ED presentation**		
7:00 a.m. to 2:59 p.m.	440,000	40.1 (32.8–47.9)
3:00 p.m. to 10:59 p.m.	396,000	36.1 (28.9–44.0)
11:00 p.m. to 6:59 a.m.	261,000	23.8 (17.3–31.9)
**Geographic region**		
Northeast	183,000	16.4 (9.3–27.4)
Midwest	213,000	19.1 (12.1–28.9)
South	524,000	47.0 (32.5–62.1)
West	194,000	17.4 (10.2–28.0)
**Section B. Emergency department clinical information**		
Arrival by ambulance	844,000	77.1 (59.0–88.8)
Number of comorbid conditions, mean*	1.8	(1.4–2.2)
**Most common chief complaints**		
Shock/consciousness disturbance	112,000	10.0 (5.6–17.3)
Chest pain	94,000	8.5 (4.8–14.6)
Shortness of breath	37,000	3.3 (1.5–7.1)
**Triage level**		
1	410,000	54.2 (42.8–65.1)
2	146,000	19.3 (12.6–28.4)
3	137,000	18.0 (11.8–26.5)
4	37,000	4.9 (1.0–20.6)
5	27,000	3.6 (1.6–7.8)
Body temperature, mean,°C	36.5	(36.2–36.7)
Heart rate, mean, beats per min	89.8	(83.5–96.2)
Respiratory rate, mean, breaths per min	18.5	(15.4–21.6)
Oxygen saturation, mean,%	85.8	(78.4–93.1)
Systolic blood pressure, mean, mmHg	117.9	(106.1–129.7)
Length of ED stay, mean, hours	6.6	(1.6–11.5)
**ED disposition**		
Admission	490,000	44.0 (30.4–58.5)
Died in the ED	564,000	50.7 (37.8–63.4)
**Hospitalization**		
Length of hospital stay, mean, days	6.2	(3.7–8.6)
Inpatient mortality	155,000	51.6 (33.9–68.8)

*ED, emergency department.*

*Available from 2012 to 2018.

**FIGURE 2:**
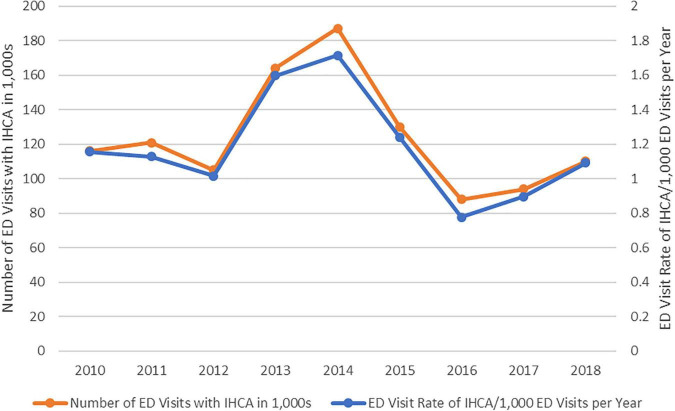
The number and incidence (visit) rate of emergency department-based in-hospital cardiac arrest, 2010–2018.

Figure 2 shows the number and incidence rate of ED-based IHCA from 2010 to 2018. The number of ED visits with IHCA ranged from 80,000 to 200,000 patients. The incidence rate of ED-based IHCA ranged from 0.6 to 1.8 in 1,000 annually (P for trend = 0.49), and the trend paralleled that of ED-based IHCA number each year. Table 2 shows the factors associated with the incidence of ED-based IHCA. The overall ED IHCA rate was 1.2 per 1,000 visits (95% CI, 0.9–1.6/1,000 visits). Elderly patients had a significantly higher risk of ED-based IHCA (5.0–5.9 times higher), and the trend increased with age (from 0.3 per 1,000 visits to 2.6–2.7 per 1,000 visits). The incidence of ED-based IHCA was 1.8 per 1,000 ED visits in males, a 2.5 times higher risk of IHCA after adjusting for other factors. Uninsured patients had an approximately 2-fold higher risk of ED-based IHCA than those with private insurance. No differences in IHCA incidence were noted by season, weekend, time of ED presentation, or geographic region. The incidence rate of patient arrival to the ED by ambulance was 4.9 per 1,000 visits, which was higher than those who arrived without an ambulance (0.3 per 1,000 visits). In multivariable analysis, patients who arrived by ambulance had a 12 times higher odds of ED-based IHCA than those who did not. The alternative Poisson model yielded almost identical incidence rate ratio results (Supplementary Table 1). A more clinical model also showed a similar set of predictors with the addition of higher triage levels, high pulse rate, and low oxygen saturation (Supplementary Table 2).

**TABLE 2:** Emergency department in-hospital cardiac arrest visit rates, overall, stratified, and multivariable analysis, 2010–2018.

Variable	ED IHCA rate per 1,000 visits (95% CI)	Adjusted OR (95%CI)*
**Overall**	**1.2 (0.9**–**1.6)**	
**Age group**		
18–24	0.3 (0.2–0.6)	1.0 (reference)
25–44	0.6 (0.4–0.9)	1.3 (0.6–2.5)
45–64	1.4 (1.0–1.9)	**3.5 (2.0**–**6.4)**
65–74	2.7 (1.8–4.0)	**5.9 (3.0**–**11.5)**
75 +	2.6 (1.7–4.1)	**5.0 (2.4**–**10.6)**
**Sex**		
Male	1.8 (1.3–2.4)	**2.5 (1.8**–**3.5)**
Female	0.7 (0.5–1.1)	1.0 (reference)
**Race/ethnicity**		
Non-Hispanic white	1.0 (0.8–1.3)	1.0 (reference)
Non-Hispanic black	1.3 (0.8–1.9)	1.3 (0.8–2.1)
Hispanic	1.7 (0.6–4.9)	2.3 (0.6–8.6)
Other	1.6 (0.6–4.3)	2.1 (0.8–5.2)
**Insurance**		
Private insurance	0.6 (0.4–1.0)	1.0 (reference)
Medicare	2.2 (1.5–3.3)	1.0 (0.6–1.9)
Medicaid or state-based program	0.6 (0.4–1.0)	0.9 (0.5–1.6)
Self-pay (uninsured)	1.4 (0.9–2.1)	**2.2 (1.2**–**4.0)**
Other	1.1 (0.4–2.7)	1.1 (0.4–2.9)
**Season**		
Spring (Mar. – May)	1.2 (0.6–2.3)	1.4 (0.7–2.8)
Summer (Jun. – Aug.)	0.9 (0.6–1.5)	1.0 (reference)
Fall (Sep. – Nov.)	1.5 (1.1–2.2)	1.5 (0.8–2.8)
Winter (Dec. – Feb.)	1.1 (0.7–1.5)	1.1 (0.6–2.0)
**Weekend**		
Non-weekend	1.3 (0.9–1.7)	1.0 (reference)
Weekend	0.9 (0.7–1.4)	0.8 (0.5–1.2)
**Time of ED presentation**		
7:00 a.m. to 2:59 p.m.	1.1 (0.8–1.6)	1.1 (0.8–1.6)
3:00 p.m. to 10:59 p.m.	1.0 (0.7–1.4)	1.0 (reference)
11:00 p.m. to 6:59 a.m.	1.8 (1.2–2.7)	1.5 (0.9–2.4)
**Geographic region**		
Northeast	1.1 (0.6–2.0)	1.0 (reference)
Midwest	1.0 (0.7–1.4)	1.0 (0.5–2.2)
South	1.4 (0.8–2.5)	1.7 (0.7–3.9)
West	1.0 (0.6–1.5)	1.0 (0.4–2.1)
**Arrival mode**		
Arrival not by ambulance	0.3 (0.1–0.8)	1.0 (reference)
Arrival by ambulance	4.9 (4.0–6.1)	**12.0 (4.5**–**32.2)**

*Significant odds ratios are highlighted in bold.*

*ED, emergency department; IHCA, in-hospital cardiac arrest; OR, odds ratio.*

*Multivariable model adjusts for all variables in the Table.

**FIGURE 3:**
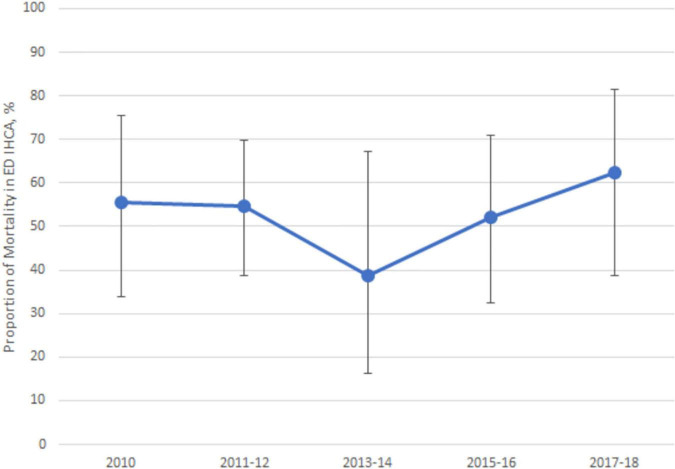
The emergency department mortality rate of in-hospital cardiac arrest, 2010–2018.

Figure 3 shows the mortality rate of ED-based IHCA from 2010 to 2018. The average mortality rate was 50%, ranging approximately from 40 to 60% during the study period (P for trend = 0.68). Table 3 displays the factors associated with mortality in ED patients with IHCA. No differences in IHCA mortality were found by age, sex, race/ethnicity, insurance status, and seasons. ED patients had higher IHCA mortality rates during a weekend visit (72%), a 3.3 times higher odds of mortality than those visiting on weekdays. The ED-based IHCA mortality rate was significantly higher at night (63%) than during the day or evening (45–48%). Hospitals in the Northeast had the lowest ED IHCA mortality rates (29%) and were independently associated with a lower mortality rate in multivariable analysis. Arrival by ambulance was also an independent predictor of ED mortality following IHCA. A more clinical model showed only higher body temperature as a single protective predictor of ED mortality after IHCA (Supplementary Table 3).

**TABLE 3:** Emergency department in-hospital cardiac arrest mortality rates, overall, stratified, and multivariable analysis, 2010–2018.

Variable	ED IHCA mortality rate,% (95% CI)	Adjusted OR (95% CI)*
**Overall**	**50.7 (37.8**–**63.4)**	
**Age group**		
18–24	55.1 (23.0–83.5)	1.0 (reference)
25–44	49.6 (29.6–68.0)	1.1 (0.1–8.8)
45–64	44.4 (27.7–62.5)	0.5 (0.1–2.9)
65–74	50.4 (30.3–70.4)	1.3 (0.2–8.5)
75 +	59.8 (34.6–80.7)	1.6 (0.2–11.4)
**Sex**		
Male	52.3 (38.7–65.7)	0.8 (0.3–1.7)
Female	47.6 (30.2–65.5)	1.0 (reference)
**Race/ethnicity**		
Non-Hispanic white	58.5 (45.6–70.4)	1.0 (reference)
Non-Hispanic black	57.0 (40.0–72.5)	1.7 (0.6–5.4)
Hispanic	23.2 (5.9–59.1)	0.3 (0.1–1.6)
Other	31.9 (8.1–71.3)	0.1 (0.0–1.6)
**Insurance**		
Private insurance	42 (23.7–62.7)	1.0 (reference)
Medicare	54 (35.5–71.3)	1.1 (0.3–3.5)
Medicaid or state-based program	49 (28.3–70.3)	1.4 (0.3–6.5)
Self-pay (uninsured)	63 (38.0–82.3)	2.3 (0.6–9.1)
Other	27 (6.5–65.9)	0.1 (0.0–2.3)
**Season**		
Spring (Mar. – May)	44.1 (17.9–74.1)	0.8 (0.3–2.2)
Summer (Jun. – Aug.)	51.4 (33.2–69.2)	1.0 (reference)
Fall (Sep. – Nov.)	49.3 (35.1–63.6)	0.7 (0.2–2.1)
Winter (Dec. – Feb.)	60.0 (41.8–75.9)	1.3 (0.4–3.6)
**Weekend**		
Non-weekend	44.7 (32.3–57.9)	1.0 (reference)
Weekend	72.2 (51.2–86.5)	**3.3 (1.2**–**8.9)**
**Time of ED presentation**		
7:00 a.m. to 2:59 p.m.	45.6 (30.0–62.1)	1.0 (0.4–2.8)
3:00 p.m. to 10:59 p.m.	47.8 (33.1–62.9)	1.0 (reference)
11:00 p.m. to 6:59 a.m.	62.8 (39.2–81.5)	**3.7 (1.1**–**12.3)**
**Geographic region**		
Northeast	29.2 (15.6–48.0)	1.0 (reference)
Midwest	58.5 (39.2–75.5)	**6.4 (1.9**–**20.9)**
South	53.7 (29.3–76.5)	**3.0 (1.1**–**8.7)**
West	54.0 (33.7–73.1)	**23.0 (4.0**–**133.5)**
**Arrival mode**		
Arrival not by ambulance	24.8 (7.4–57.9)	1.0 (reference)
Arrival by ambulance	58.8 (49.0–68.0)	**3.9 (1.2**–**12.8)**

*Significant odds ratios are highlighted in bold.*

*ED, emergency department; IHCA, in-hospital cardiac arrest; OR, odds ratio.*

*Multivariable model adjusts for all variables in the table.

## Discussion

In the current study, we found that the incidence rate of ED-based IHCA was approximately one in 1,000 ED visits in the US from 2010 to 2018. This trend appeared stable over the 9-year study period. The elderly, males, uninsured patients, and arrival by ambulance were independent risk factors for ED-based IHCA. Weekend or nighttime arrival, geographic region, and arrival by ambulance were independently associated with mortality in patients with ED-based IHCA.

### Stable Incidence and Mortality Rate of Emergency Department-Based In-Hospital Cardiac Arrest Between 2010 and 2018

The incidence rate of ED-based IHCA was relatively stable during the study period. Additionally, the number of reported IHCA was smaller than the combined number of CA from a previous study by Valderrama (1.7 per 1,000) (10). Evidence suggests an increasing number of overall IHCA (2); however, the number of ED-based IHCA appeared stable, probably due to its relatively small proportion in overall IHCA. Moreover, the trend in mortality was relatively stable over the 9-year study period. Half of the patients in this study died in the ED after resuscitation. Furthermore, half of the patients who were admitted to the hospital died in the hospital eventually. Advances in critical care medicine have led to a slight decrease IHCA mortality rates (inpatient mortality) over time, but this survival benefit appeared to have plateaued (5, 6). These observations were consistent with our findings of no changes in rates of ED-based IHCA mortality. The barrier to further improvement may be a lack of new therapeutic agents for IHCA (16).

### Factors Associated With a Higher Incidence Rate of Emergency Department-Based In-Hospital Cardiac Arrest

In this study, the baseline characteristics of patients with ED-based IHCA (e.g., older age, male) were similar to those reported in a previous study using the NHAMCS data, 2001–2007 (10). Additionally, uninsured patients independently predicted ED-based IHCA. These patients may have poor health conditions, lower socioeconomic levels, and difficulties accessing health care, all of which may have increased the risk of ED-based IHCA (17–19). Similarly, there was a higher IHCA incidence among pediatric patients with lower median household incomes (20). Taken together, these findings outline the patient populations that are prone to IHCA in the ED. These factors are consistent with key items in the predictive tool for ED-based IHCA that we previously developed (21).

### Different Patterns of Circadian Variation in Emergency Department-Based In-Hospital Cardiac Arrest (Incidence vs. Mortality)

We did not observe circadian variations in ED-based IHCA incidence rates. Several previous studies have examined the circadian pattern of cardiac arrest, but the findings remain inconsistent. OHCA occurrences appeared to have a morning peak (22–24); however, IHCA does not seem to have a circadian pattern (25). By contrast, in this study, ED-based IHCA mortality rate was highest at night. Furthermore, patients with ED-based IHCA on the weekend had lower survival rates than on weekdays. Previous reports have indicated lower survival-to-hospital discharge rates in patients with IHCA on the weekend (26, 27). Another report has suggested that IHCA during the midnight shift was independently associated with a lower probability of return of spontaneous circulation compared with IHCA occurring during daytime (28). This may result from fewer health care providers at midnight, which may, in turn, lead to compromised resuscitation team efforts and poorer resuscitation quality.

### Emergency Department -Based In-Hospital Cardiac Arrest Mortality Rates Differed by Geographic Region

Our study demonstrated that ED-based IHCA visit rates did not differ significantly by geographic region, consistent with a prior study between 2001 and 2007 (10). However, we found regional variation in ED IHCA mortality rates, which was not previously reported. One early population-based study from the Nationwide Inpatient Sample database analyzed adult IHCA patients from 2003 to 2011. These data indicated the highest and lowest incidence in the West and Midwest regions, respectively. The Northeast region had the highest inpatient mortality when compared with other regions (29). Conversely, in our study using more recent data, the Northeast region had the lowest ED-based IHCA mortality rate compared with other regions. Furthermore, the West had the highest ED-based IHCA mortality rate after 2010. Because some factors known to be related to IHCA outcomes are not available in the NHAMCS (e.g., shockable rhythm and CPR duration), the regional variation in ED-based IHCA mortality may be due to differences in case-mix and resuscitation practices (30, 31).

### Limitations

This study has some potential limitations. First, we used “dead on arrival” (DOA) to exclude patients with OHCA. However, not all DOA patients had CPR marks on their PRF (i.e., ongoing CPR on ED arrival). It is possible that some OHCA patients were still included in our analysis. Second, our study focused on ED-based IHCA outcomes, namely, ED mortality (survival-to-hospital admission). The NHAMCS did provide data on inpatient mortality (survival-to-hospital discharge); however, the case numbers were too low to perform a meaningful analysis. Also, we did not compare ED-based IHCA vs. non-ED-based IHCA. Third, although the NHAMCS collected a wealth of information, some detailed clinical Utstein-style variables were lacking from the NHAMCS, such as initial cardiac rhythm, time to resuscitation, resuscitation duration, inotropes use, event witnessed, and defibrillation. These factors could alter ED mortality rates. Finally, this is a retrospective study and the associations identified are not necessarily causal. Therefore, the associations identified may be subject to missing data or uncollected/unmeasured potential confounders and need to be confirmed in future studies.

## Conclusion

In this 9-year study representing approximately 1 million IHCA ED visits, the incidence of ED-based IHCA (1.2 per 1,000 ED visits) appeared relatively stable. The medical burden remained high, and the survival-to-hospital discharge rate was poor. The factors associated with ED-based IHCA were older age, male, uninsured status, and arrival by ambulance. The factors related to ED mortality included time of presentation to the ED, geographic region, and arrival by ambulance. The patient factors found in our study could help identify patients at a higher risk of IHCA in the ED. The contextual factors associated with outcomes could help organize a better system of care for millions of ED patients with IHCA.

## Data Availability Statement

The datasets presented in this article are not readily available because the datasets used and/or analyzed during the current study are available from the corresponding author on reasonable request. Requests to access the datasets should be directed to C-LT, chulintsai@ntu.edu.tw.

## Ethics Statement

The studies involving human participants were reviewed and approved by the National Taiwan University Hospital. The ethics committee waived the requirement of written informed consent for participation.

## Author Contributions

C-WS, T-CL, and C-LT contributed to conception and design of the study. C-HW and EC organized the database. C-HK performed the statistical analysis. C-WS wrote the first draft of the manuscript. C-HH, W-JC, and C-LT wrote sections of the manuscript. All authors contributed to manuscript revision, read, and approved the submitted version.

## Conflict of Interest

The authors declare that the research was conducted in the absence of any commercial or financial relationships that could be construed as a potential conflict of interest.

## Publisher’s Note

All claims expressed in this article are solely those of the authors and do not necessarily represent those of their affiliated organizations, or those of the publisher, the editors and the reviewers. Any product that may be evaluated in this article, or claim that may be made by its manufacturer, is not guaranteed or endorsed by the publisher.
