# Supplementary material for: In-Hospital Cardiac Arrest in United States Emergency Departments, 2010–2018
Source: Front Cardiovasc Med. 2022 Apr 11;9:874461. doi: 10.3389/fcvm.2022.874461 (PMC9035594; doi:10.3389/fcvm.2022.874461)
Supplement: Supplementary file 1 [file Data_Sheet_1.docx]

**Online Supplemental Table S1.** Emergency department in-hospital cardiac arrest incidence rate ratios in multivariable analysis, 2010-2018.

| **Variable** | **Incidence Rate Ratio (95%CI)*** |
| --- | --- |
| **Overall** |  |
| Age group |  |
| 18-24 | 1.0 (reference) |
| 25-44 | 1.3 (0.6-2.5) |
| 45-64 | **3.5 (2.0-6.3)** |
| 65-74 | **5.8 (3.0-11.3)** |
| 75+ | **5.0 (2.4-10.5)** |
| Sex |  |
| Male | **2.5 (1.8-3.5)** |
| Female | 1.0 (reference) |
| Race/ethnicity |  |
| Non-Hispanic White | 1.0 (reference) |
| Non-Hispanic Black | 1.3 (0.8-2.1) |
| Hispanic | 2.3 (0.6-8.4) |
| Other | 2.1 (0.8-5.2) |
| Insurance |  |
| Private insurance | 1.0 (reference) |
| Medicare | 1.0 (0.6-1.9) |
| Medicaid or state-based program | 0.9 (0.5-1.6) |
| Self-pay (uninsured) | **2.2 (1.2-4.0)** |
| Other | 1.1 (0.4-2.9) |
| Season |  |
| Spring (Mar. – May) | 1.4 (0.7-2.7) |
| Summer (Jun. – Aug.) | 1.0 (reference) |
| Fall (Sep. – Nov.) | 1.5 (0.8-2.8) |
| Winter (Dec. – Feb.) | 1.1 (0.6-2.0) |
| Weekend |  |
| Non-weekend | 1.0 (reference) |
| Weekend | 0.8 (0.5-1.2) |
| Time of ED presentation |  |
| 7:00 am to 2:59 pm | 1.1 (0.8-1.6) |
| 3:00 pm to 10:59 pm | 1.0 (reference) |
| 11:00 pm to 6:59 am | 1.5 (0.9-2.3) |
| Geographic region |  |
| Northeast | 1.0 (reference) |
| Midwest | 1.0 (0.5-2.2) |
| South | 1.7 (0.7-3.8) |
| West | 1.0 (0.4-2.1) |
| Arrival mode |  |
| Arrival not by ambulance | 1.0 (reference) |
| Arrival by ambulance | **12.0 (4.4-32.1)** |

Significant odds ratios are highlighted in bold.

Abbreviations: ED = emergency department; CI = confidence interval.

*Multivariable model adjusts for all variables in the Table.

**Online Supplemental Table S2.** Emergency department in-hospital cardiac arrest visit rates in multivariable analysis, 2010-2018.

| **Variable** | **Adjusted OR (95%CI)*** |
| --- | --- |
| **Overall** |  |
| Age, per 1-year increase | **1.028 (1.008-1.048)** |
| Sex |  |
| Male | **1.9 (1.1-3.5)** |
| Female | 1.0 (reference) |
| Insurance |  |
| Private insurance | 1.0 (reference) |
| Medicare | 1.4 (0.5-3.9) |
| Medicaid or state-based program | 1.6 (0.5-5.4) |
| Self-pay (uninsured) | **3.0 (1.1-7.9)** |
| Other | **5.1 (1.5-17.4)** |
| Triage level |  |
| 1 | **23.9 (9.7-58.8)** |
| 2 | **2.1 (1.05-4.25)** |
| 3 (reference) | 1.0 |
| 4 | 0.7 (0.1-3.4) |
| 5 | 0.9 (0.2-5.9) |
| Triage vital signs |  |
| Temperature | 0.7 (0.4-1.1) |
| Heart rate | **1.02 (1.002-1.031)** |
| Respiratory rate | 1.00 (0.95-1.05) |
| Systolic blood pressure | 0.99 (0.97-1.01) |
| Oxygen saturation | **0.97 (0.95-0.98)** |
| Arrival mode |  |
| Arrival not by ambulance | 1.0 (reference) |
| Arrival by ambulance | **6.7 (2.9-15.5)** |

Significant odds ratios are highlighted in bold.

Abbreviations: OR = odds ratio; CI = confidence interval.

*Multivariable model adjusts for all variables in the Table.

**Online Supplemental Table S3.** Emergency department in-hospital cardiac arrest mortality rates in multivariable analysis, 2010-2018.

| **Variable** | **Adjusted OR (95%CI)*** |
| --- | --- |
| **Overall** |  |
| Age, per 1-year increase | 1.013 (0.936-1.097) |
| Sex |  |
| Male | 6.0 (0.6-58.3) |
| Female | 1.0 (reference) |
| Triage level |  |
| 1 & 2 | 1.9 (0.3-13.7) |
| 3 (reference) | 1.0 |
| 4 & 5 | 1.5 (0.007-319.4) |
| Triage vital signs |  |
| Temperature | **0.1 (0.02-0.71)** |
| Heart rate | 1.03 (0.97-1.10) |
| Respiratory rate | 1.05 (0.89-1.23) |
| Systolic blood pressure | 0.98 (0.96-1.01) |
| Oxygen saturation | 1.2 (0.93-1.65) |
| Arrival mode |  |
| Arrival not by ambulance | 1.0 (reference) |
| Arrival by ambulance | 0.4 (0.07-2.43) |

Significant odds ratios are highlighted in bold.

Abbreviations: OR = odds ratio; CI = confidence interval.

*Multivariable model adjusts for all variables in the Table.
